# Artificial Intelligence developments in medical education: a conceptual and practical framework

**DOI:** 10.15694/mep.2020.000239.1

**Published:** 2020-10-26

**Authors:** Ken Masters

**Affiliations:** 1 Sultan Qaboos University

**Keywords:** Artificial Intelligence, Medical Education

## Abstract

This article was migrated. The article was marked as recommended.

As Artificial Intelligence (AI) develops in medicine, there is a greater awareness that medical education may also benefit from AI. Many AI projects are already underway, and many more are still to come. Most medical education administrators and educators are aware of AI, but are not necessarily familiar enough with it to understand the areas of possible application in both using AI in medical education, and also in the content areas that need to be raised with their students. As such, they are at a disadvantage of not understanding the current lie of the land, and are at an even greater disadvantage of not being able to influence and guide future AI projects. This paper attempts to provide an AI conceptual and practical framework for medical education administrators and educators, so that they may have a clearer understanding of the current situation, and may be better placed to guide future AI developments to meet their needs in medical education.

## Introduction

As a central part of the Fourth Industrial Revolution ( Schwab, 2015), developments in Artificial Intelligence (AI) currently indicate that AI will have a great impact on all fields of human knowledge and activity. The impact of AI in *medicine* is well-covered in the literature; as this journal focuses on *medical education*, however, there is nothing to be served by recounting the history and status of AI in *medicine*: all indications are that it will eventually arrive. But just what “it” will look like in *medical education*, how it will affect our students, and how best to harness its strengths, is currently not entirely clear.

What *is* clear, however, is this: if we wish AI in medical education to meet our needs, then we should clearly identify and understand those needs, ground them carefully in medical education principles, and have a hand in guiding the development of the projects so that they really do meet our needs.

The *AMEE Guide to AI in Medical Education* ( Masters, 2019) has given a broad overview of possible areas, and also touched on a few topics in detail. Since then, there have been various publications on AI in medical education (e.g. (van der Niet and Bleakley, 2020), and many research projects (described in a little more detail below), but, as yet, there does not appear to be a coherent framework for medical schools to use as a context into which they may place, or through which they may assess, these projects. This is made more complex by the fact that, internationally, medical schools have several different models of student intake, and some students arrive fresh from high school, while others already have a degree, possibly in engineering or computer science.

This paper attempts to create a broad framework that can be adopted by medical schools, irrespective of their student intake or their education model. It presents the overall framework and describes the various components within that framework, and the issues raised. It is specifically aimed at non-AI experts, and so it has very limited technical jargon, focussing on medical education concerns. The resultant framework will allow medical schools to assess their own position in relation to AI projects, to place these projects within that framework so as to better understand them, and then to further develop new projects based upon their needs.

## The Framework

The Framework contains two broad areas of medical education in which AI can be used: *Administration &* *Methodology* and *Content.*

The *Administration &* *Methodology* area falls under the broader umbrella of Knowledge Management and Knowledge Application, and covers the education mechanics and processes including administrative, supportive and pedagogic.

The *Content* area focuses on the material that is to be learned and understood by medical students in order to practice medicine in a world of AI.

**Figure 1.**
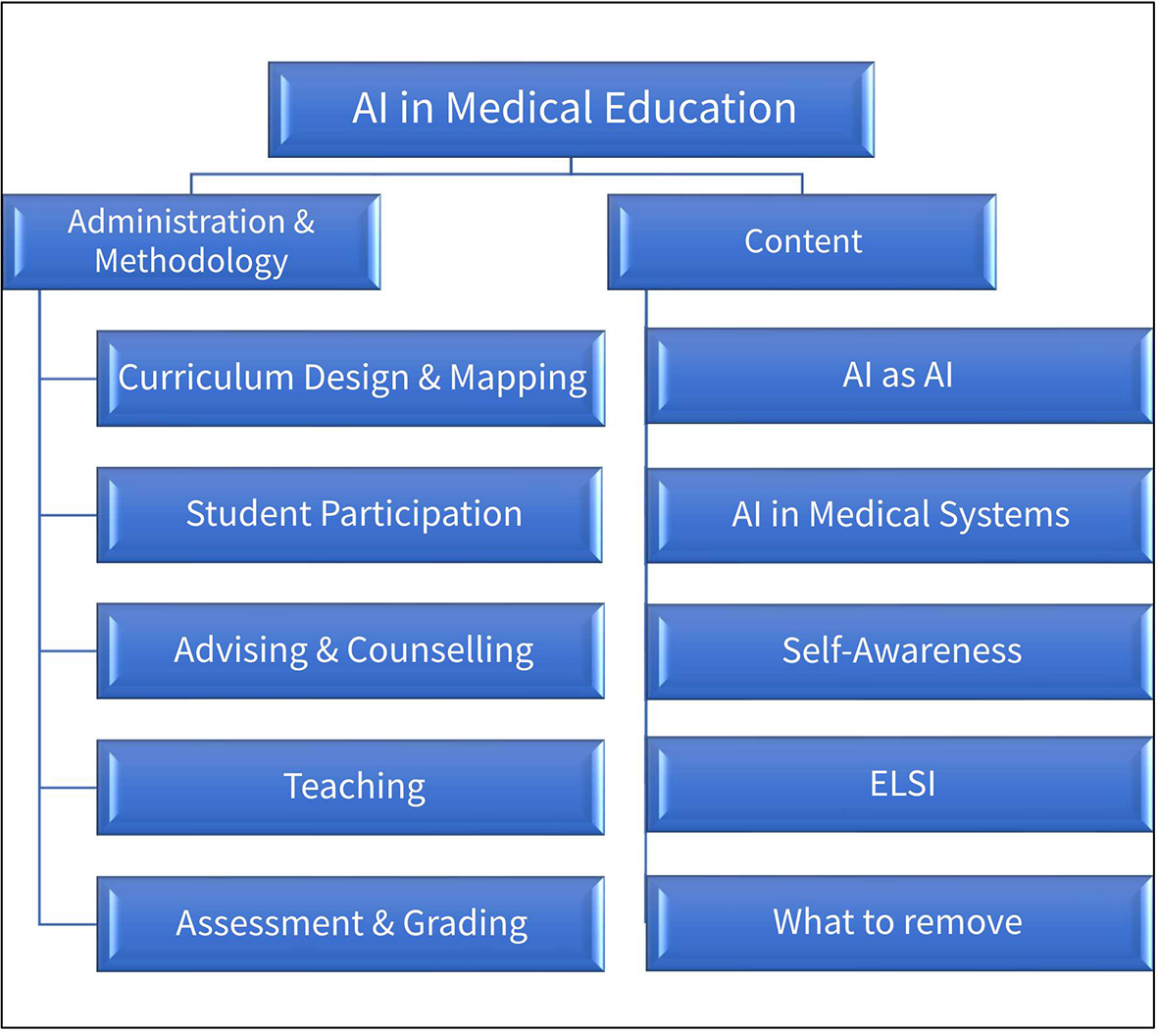
Schematic representation of issues to be covered in AI in Medical Education.

Each of these areas contain sub-topics. Schematically, they can be represented as Figure 1.

Before we examine these in detail, it should be noted that it is unlikely that medical schools will have the expertise to implement all of these and simultaneously. It will probably be necessary for medical schools to collaborate closely with other schools, and also with other subject areas, such as engineering and computer science, and to tread slowly.

## Administration & Methodology

The complexities of curriculum mapping, especially in medical education, are well-documented ( Harden, 2001). As Harden notes, the key functions of curriculum mapping are to make the curriculum more transparent to all stake holders (students, teachers, and managers), and also to show how all the elements are inter-connected. Without a curriculum map, we simply do not know where we are, where we have come from, or where we are going.

Although creating a curriculum map is exhausting and time-consuming, that is not the real difficulty. The real difficulty is keeping the map current as the curriculum is re-designed and changed, and also as the map becomes applicable differently to different learners and teachers. Just as a 10-year old map of a country and its roads is inaccurate for the traveller today, so a curriculum map that is outdated is also useless, or worse, harmful, as we make decisions based on out-dated information.

For example, as a medical teacher, even if I do have a current curriculum map of my medical school, it is a generic snapshot, showing only today (or since the committee last updated it), and for this student “cohort”. If I am teaching a class of 5 ^th^-year medical students, I do not need to know what the current 3 ^rd^-year students are studying; I need to know *what these 5 ^th^-year students studied when they were in 3 ^rd^-year.* More specifically, I need to know what Jane Doe studied when *she* was in 3 ^rd^-year, irrespective of when or where she did it, or repeated a module, semester or year, or took a leave of absence, or was granted an extension.

Similarly, during clinical training, which rotations have been completed by this student, when and at which centre?

The moment I want *that* information, the generic map proves inaccurate and harmful, and then I have to resort to mounds of other documents and transcripts. In short, we need a Google Maps ^TM^ for our curriculum - not only showing the land as it is now, but showing where each traveller is and the journey they have undertaken and the road ahead. And, as the environment changes (a new course, a new teacher, new material), the map has to be automatically updated to reflect these changes.

One of the reasons that current curriculum maps cannot do this is because they are designed for the institution. An AI-generated curriculum map, rather than centred on the institution, would be centred on the needs of the learner and the teacher, and cover all their work, *backward and forward in time.* It then would go further and deeper, and include direct access to the materials used by the student, grades, assignments, etc. Thus, the curriculum map becomes the central tool, not only for the organisation, but also for materials access, augmenting (or even replacing), the current-style Learning Management System (LMS).

We will, therefore, need AI to develop curriculum maps that fulfil Harden’s vision of linking all of the stake holders, dynamically, in a continually-changing environment, and tie these to the tracking information currently contained in transcripts and course blue-prints, and even down to document level, so that the teacher of the 5 ^th^-year student can find out what Jane Doe studied in 3 ^rd^-year, and locate the PowerPoint presentation that was given to her and assignments she completed (as opposed to another student who was in a different cohort).

There will also need to be portability, so that a student can move from or to other institutions, or incorporate courses from other institutions, and these would automatically be mapped into that student’s curriculum map. This would naturally affect the notion of a “student cohort” as individual students move through a curriculum with flexibility of time and material.

By referring to flexibility of material, I am not saying that each student chooses their entire degree from a smorgasbord. (Although we already attempt to cater to different backgrounds and abilities, having different tracks and choices.) I am, however, allowing for the map to be able to identify all the differences. Once all of these are clearly identified, the AI system can also perform detailed analyses and evaluations of the different curricula ( Chen, 2010).

Finally, as the map will be complex in space and time, access methods should be through immersive, virtual-reality technologies, rather than the current 2-d document approach. Who would have thought that curriculum design and mapping could be such an exciting venture?

In the past, individual instructors (and medical schools) kept records on class attendance. There is the old adage that, if you’re concerned about counting bums on seats, then you’re worried about the wrong end of the student. While there are many studies showing correlations between attendance and grades ( Ullah *et al.*, 2020), one should not be tempted into an over-simplification of causality that simply attending class will increase grades, as a host of other factors, especially direct participation, are at play, although many of these can be, and are being, measured ( Jović *et al.*, 2019).

One can understand the teacher’s needs for monitoring, because there simply is no real method to accurately measure the impact of organised activities, and teachers simultaneously wish to identify at-risk students before problems become so large that they are insurmountable.

The more one measures, however, the more one raises privacy concerns, and we need to ensure that we are measuring something worth measuring, and interpreting the measurements correctly. For example, while using AI facial recognition technology to determine (and grade) student attention ( Conner, 2018) may have “positive” results, and brain monitoring through other devices such as FocusEDU ( https://www.brainco.tech/) appears desirable, this Orwellian dystopia does not directly address our pedagogical concern of ensuring the participation of the student in the learning process, and leading to the well-prepared doctor. Perhaps somewhat less disturbing for students is the use of eye-tracking ( Alemdag and Cagiltay, 2018; Ashraf *et al.*, 2018), which can also be used to give feedback information about the effectiveness of the system at focusing the student’s attention, although this may also be perceived as invasive.

For online learning, most LMSs can track student attendance and participation, especially on obvious items like files downloaded, forum posts read and made, and various manual or automatic alerts can be created so that corrective action can be taken. Some systems already use various simple AI models for predicting at-risk students by combining grades, participation and other activities, something that is made far more possible because of students’ digital presence ( Hershkovitz *et al.*, 2013; Babić, 2017; Hoffait and Schyns, 2017; Hussain *et al.*, 2018; Toivonen, Jormanainen and Tukiainen, 2019; Bernacki, Chaves and Uesbeck, 2020). These are currently reasonably intuitive, although they, and other measures (e.g. student satisfaction), do require careful interpretations, and educators need to ensure that they apply reasonable educational principles rather than have an automatic response to a set of parameters ( Parapadakis, 2020).

As a side note: while the focus of this topic is on the course, similar AI techniques can be applied across the Internet to determine what current students think of the medical school as a whole ( Srinivas and Rajendran, 2019).

Not all student participation has to be monitored in this way; student feedback is a crucial aspect of education. The prime difficulty is being able to analyse the student feedback data so that appropriate responses can be undertaken. Current investigations into AI analysing student feedback of both instructors and courses ( Agaoglu, 2016; Rani and Kumar, 2017; Zhang, Zhang and Xu, 2019) give an indication of possibilities (not only of revising the courses, but also the (tired and outdated) feedback tools).

As with all systems, the checkboxes can be circumvented (or “gamed”), and there is a responsibility on the student: if a student games the system, and all teachers believe that all is well with the student, then, by the time the truth of the situation emerges, it will be too late for any intervention to have a positive impact, and the student will suffer.

Prior to enrolment, systems can be used to predict student performance, and, as a bonus, prediction modelling can sometimes show prejudices and weaknesses in enrolment acceptance policies and practices ( Andris, Cowen and Wittenbach, 2013; Alban and Mauricio, 2019).

Academic advising is frequently complicated by a bureaucratic swamp of rules and choices, and AI needs to ensure that all pathways are obvious to all stakeholders. In much the same way that Clinical Decision Support Systems are used by doctors to advise patients, so Academic Advice Support Systems need to be developed to assist academic advisors when advising students, and work in this field has already shown promise ( Kardan and Sadeghi, 2013).

These systems do not always have to be controlled by the administrators and academic advisors, however, and students should be able to harness the AI system themselves, usually through chatbots, which are still in their infancy and require high-end processing power, but various experiments across languages show potential for widespread use ( Khin and Soe, 2020).

In this way, we can offer far greater assistance to students when they are applying for courses ( Acikkar and Akay, 2009) and more easily identify at-risk students ( Oancea, Dragoescu and Ciucu, 2013; Asogwa and Oladugba, 2015). Again, this system can be a part of the curriculum mapping system, monitoring the student in real-time, and adjusting predictions and warnings as more data become available.

The idea of electronic tutors has existed for at least 40 years ( Clarke, 1980). Intelligent medical education tutoring systems, with adaptive instruction and testing and feedback, even in Continuing Medical Education (CME) ( McFadden and Crim, 2016) have also existed for a long time, although early systems generally required a great deal of computing expertise to use them properly, and were not very intuitive for the average medical educator ( Clancey, 1983; Papa and Shores, 1992; Eliot and Woolf, 1995; Billinghurst and Savage, 1996; Bourlas *et al.*, 1996; Sharples *et al.*, 1997; Frize and Frasson, 2000; Stasiu *et al.*, 2001; Michael *et al.*, 2003; Weidenbach *et al.*, 2004; Randhawa and Jackson, 2020).

The promise of AI lies in personalising the teaching, responding to special needs, whether activated by performance data, or specifically requested by the student. Pre-instruction assessment of needs and capabilities should be used to tailor the material and teaching methods as required. There are many research projects that give an indication of how this is already being done ( Chi *et al.*, 2011; Chaudhri *et al.*, 2013), and the process would be strongly enhanced by using the type of personal curriculum-mapping outlined above, otherwise there is the risk that the personalised tutor is an add-on, rather than an integral component in the entire system.

In addition, the tutoring system should not merely be a knowledge-transfer system, but one designed on current educational principles such as cased-based, problem-based, and complex scenario-based gamification that can move beyond managing a patient with a single condition under contextless circumstances. Interaction with the student can utilise Natural Language Processing (NLP) ( Chary *et al.*, 2019), and these systems can then present flexible and realistic virtual patients, constructed by AI, based upon real patient records ( Wijayarathna and Zary, 2019; Afzal *et al.*, 2020), requiring critical thinking and clinical reasoning, and adjusting the levels of complexity to better suit the level at which the student is expected to perform. One can then have effective virtual (or even robotic) patient case simulation for general use ( Hayasaka, Fujikura and Kashimura, 2018; Laleye *et al.*, 2020) or within specific environments, such as single or multi-centre PBL ( Caudell *et al.*, 2003; Hamdy *et al.*, 2017).

The overall goal is a move away from paper cases, by using interactive holographic virtual patients in a 3-d environment, and longer and complex scenarios, using adaptive serious games. Variations can be assigned by adjusting variables (randomly, but realistically limited by parameters), and generating new problems based on the data. In student-driven learning, the student should be able to adjust these also, to assess their ability to cope with a range of extra complexities. (This does not have to be PBL, but can be in any teaching environment). The impact of AI is not only on the delivery of the cases, but also in analysis of the collaboration, solving several education problems, including that of having passengers in the group ( Jović *et al.*, 2019).

While the vision of the electronic tutor may frequently be focused on undergraduate theoretical teaching, there are several useful projects that utilise AI or intelligent machine tutors specifically in clinical skills teaching (e.g. ( Yang and Shulruf, 2019; Bakshi *et al.*, 2020; Mirchi *et al.*, 2020; Treceño-Fernández *et al.*, 2020; D. Wu *et al.*, 2020). Currently, the focus of AI in e-learning systems appears to be on the use of Bayesian Networks and Artificial Neural Networks ( Jović *et al.*, 2019). This networking allows the system to learn from the student and other students, and flexibly adapt its methods and materials as the student grows and changes. When AI teaching is combined with robotics and physical simulations, we then have a physical AI robotic simulated patient ( Hayasaka, Fujikura and Kashimura, 2018).

After the student has qualified, other types of AI tutoring systems will be required, as CME requires so much more self-organisation and self-discipline, so the system should more closely resemble a personal advisor and tutor that constantly monitors research areas and constructs courses tailored to the individual professional.

Currently, wide-spread computer-based theoretical assessment is performed through MCQs, with seemingly very little advancement past that level. The needed aim of AI is to produce meaningful assessments that allow for personalisation of format, content, and provision of additional materials as is required, so that all forms of assessment, including formative, can be enhanced. These developments would need to be linked to the developments of the flexible curriculum map outlined above.

Two examples of areas that may be of particular interest to medical educators are:

• Essays. While the value of longer, written assessment pieces is acknowledged, the practicalities of grading reduce their application. Currently, AI work is being done in the area ( Gierl *et al.*, 2014; García-Gorrostieta, López-López and González-López, 2018), although there is still a long way to go, as some industry tools appear to be based on simplistic key-word technology, and this type of grading will require highly sophisticated language and context analysis ( Kintsch, 2002; Latifi *et al.*, 2016). There are already related models for tasks like predicting the chances of journal paper acceptance ( Skorikov and Momen, 2020), although these are still small and very focused.
• Surgery. In these models, motion tracking is applied to examination of surgical procedures, and realistic student assessment can be performed ( Oquendo *et al.*, 2018; Alonso-Silverio *et al.*, 2018; Anh, Nataraja and Chauhan, 2020; Macrae *et al.*, 2020).

We need to be aware that assessment can be incorporated into almost any AI tutorial system. Earlier, mention was made of eye-tracking systems, and these can also be used for overall assessment, including in clinical procedures ( Ashraf *et al.*, 2018). With a few changes to almost any instructional module, it can be adapted to pause, wait for student input, respond to that input, and then continue. Further adaptation on wrong-answer analysis, and group-participation analysis will add extra value to the assessment.

## Content to be Taught

The paper now switches to the second large area in the framework: the content to be taught to medical students.

In the Introduction, mention was made of the range of student backgrounds when entering medical school, and it is particularly important that each medical school adjust their curriculum to allow for these backgrounds. Exactly how that would be done would be unique to each school, so is beyond the scope of this paper, but there are some guiding principles and ideas.

Teaching medical students about AI will be a requirement and will need to be preceded by a detailed analysis of the students’ previous exposure to computing and AI.

At the very least, students with little or no exposure to AI will require some knowledge of AI. Current studies show that medical students are aware of this need, but knowledge and understanding of the issues is internationally variable ( Jindal and Bansal, 2020; Sit *et al.*, 2020). Just as we concentrated on computer literacy two decades ago, we need now to teach AI literacy and a basic understanding of Data Management and AI concepts, models and terminology (such as big data (and the growing number of Vs), data mining, machine learning, deep learning, supervised and unsupervised learning, natural language processing and neural networks). For students who are new to AI, there are several good introductory texts, such as ( National Research Council, 2015; Kolachalama and Garg, 2018; Singh, 2018). (I have purposely not explained these concepts in this article, as they are covered in these texts).

Some students, however, may already have had exposure to AI in computer science, engineering or other degrees, and so would be dismayed at having to re-do this work. For these students, the curriculum will need to be adjusted, and electives, projects dealing with AI applications in solving medical problems, and assessing AI evaluations (see below) would be a starting point.

In all cases where AI is taught, the current limitations of AI need to be identified, otherwise there is a risk that AI will be seen as a magic bullet, and we may have inappropriate levels of expectation, and suffer from “automation bias” ( Mosier and Skitka, 1996). In addition, there is always the risk associated with new systems, especially new IT systems, as users struggle to adapt to them and understand them and their limitations ( Baker, Harrison and Muir, 2006), one of which is sometimes referred to as “Artificial Stupidity”. Understanding these systems will be necessary to evaluate the applicability and appropriateness of solutions.

This understanding is also important for performing research on the effectiveness of AI: one needs to know *what* to evaluate before we can know *how* to evaluate it properly. At the moment, most evaluation of medical AI systems focuses on measuring accuracy and time, especially as opposed to humans, sometimes barely qualified, under particular and ideal circumstances (e.g. ( Nirschl *et al.*, 2018; Entezarjou *et al.*, 2020; J.T. Wu et al., 2020). That is a good start, but will soon become outdated as a benchmark, for the simple reason that medicine is already arriving at stages where AI is proving superior to humans in several aspects under ideal conditions. There will need to be other criteria and modelling that will need to be developed and used as benchmarks; these will include other AI systems and less than ideal conditions. Medical educators will need to look for these and ensure that these are incorporated into their teaching. Useful overviews and starting guides include ( Wilkinson *et al.*, 2016; Jayaraman *et al.*, 2019; Liu *et al.*, 2019; Masters, 2019).

Students will need to know the mechanics and processes of AI systems that they will be expected to use. The particular medical AI systems to be taught to students would be dependent almost entirely on the types of systems that the students are likely to encounter during their clinical training and immediately after. Some basic principles, however, apply:

• As the systems are frequently updated, teachers will have to remain abreast of developments. One only has to think of how frequently the operating system on one’s personal computer is updated to have an idea of the difficulty of staying abreast.

• When systems outside the immediate experience are chosen as examples, educators will need to be extremely careful about evaluating the systems and articles written about them, as frequently the methods of system evaluation are exploratory and not always tested under real-world conditions ( Nagendran *et al.*, 2020).

• It is crucial to teach these systems as integral to the basics, and not as add-ons, otherwise they will be seen as unimportant, and new doctors will have to learn to use them without proper training, or within a context prejudicial to their effectiveness. While there is a danger of being unthinkingly reliant on AI, ignoring it would be worse. For example, in reading ECGs, although there may be legitimate concerns about its being “common practice for clinicians to wholly consign interpretation responsibility to rather unreliable computerised ECG algorithms” ( Kashou *et al.*, 2020), the solution to this problem is to ensure that ECG training courses include using AI systems, and using them judiciously. Unless at an underserved institution, medical students completing a course that does not teach the use of AI systems in ECG interpretation would be unprepared for the modern environment in which they will have to function.

The psychological impact of AI in all people’s lives in general will be great; already anthropomorphism of simple computers is a concern, and will be even greater with AI ( Salles, Evers and Farisco, 2020). The two headings above focus on the technical and practical application and usage of AI, but good medical use of AI must not include only technical usage. There needs to be a self-awareness, in which the doctor is not merely *using* the tool, but is *engaged in a cooperative exercise with the tool.* This co-operation does not imply compliance, but rather operating together.

Although health professionals are repeatedly told that they cannot be replaced by machines, on a daily level, they see machines performing routine tasks more efficiently than they can. Even currently, without advanced AI and networked systems, app stores carry apps that replace the GP for mundane tasks ( Wattanapisit *et al.*, 2020). The future reality is not so much about AI’s replacing doctors, but rather about AI-enabled doctors’ replacing non-AI-enabled doctors. Currently, doctors occupy a relatively high professional status in society, not least of all because of their perceived intellectual superiority; when a machine can do what the doctor can do, and better, one must consider the impact on this social status. Will their intellectual superiority be shaken or even displaced?

Simultaneously, however, we also know that a large amount of health care is not about the data, but about sitting with the patient, listening, asking, and interacting through human emotions, especially empathy. At this stage, one cannot believe that mounds of data are going to tell a doctor how to best deal with a specific dying patient and their family.

This might be the opportune moment for medical schools to take stock of exactly how much emphasis they place on these qualities, as, more than 70 years ago, educators recognised how students moved from the “pre-cynical” to the “cynical” years ( Becker and Geer, 1958), and this loss of empathy has been recorded in several studies since ( Hojat *et al.*, 2009; Neumann *et al.*, 2011; Chen *et al.*, 2012). A useful exercise would be for medical professionals to reflect on their training, and ask how much teaching time was devoted to the hard “scientific” aspects, and how much teaching time was devoted to human interaction and empathy. Perhaps, when medical schools come to terms with those conflicts, they will be better prepared to teach their students about AI and Empathy, or, indeed, Artificial Empathy.

The relationship with patients will need to be addressed. With professionals who rely on AI for deeply personal interactions concerning patients, the impact will surely be greater, and students will need to have an awareness of themselves and their roles in these interactions.

Correspondingly, just as health professionals are currently adapting to e-patients’ becoming more involved in their healthcare ( Masters, 2017), so they will need to adapt to e-patients’ use of AI systems. The beginning of this will be understanding their role in relation, not only to AI, but to the AI-empowered e-patient.

Related to the health professionals’ perception of themselves and their role in healthcare, a host of Ethical, Legal and Social Implications emerge, and medical students will need to consider these and the questions they raise.

Quality treatment and patient safety will rely heavily on AI algorithms, and these algorithms will (initially, anyway) be designed by humans. The algorithms will automatically carry the ethical biases of the designers ( Straw, 2020). Medical students will need to be alerted to these potential biases and how best to respond to, and even adjust, them.

Advanced AI naturally moves away from the early simplistic algorithms, and begins to take into account the ethical considerations of the world. While this does have the advantage of ensuring that the AI’s ethics is not confined to those of the original algorithms’ designers, the world’s ethics are also complex. If any sub-set of a society has its ethical view taken as representative, then previously published AI ethical fiascos ( Vincent, 2016; O’Neill, 2017; Natarajan and Nasiripour, 2019) will be repeated; on the other hand, if a simple majority view is taken, then ethics will be based on a fluctuating mob-rule mentality.

In addition, because AI is so reliant upon data, and we know that some large medical datasets have an over-representation of some groups ( Gijsberts *et al.*, 2015), these could also influence biases in AI ( Parikh, Teeple and Navathe, 2019). Given the recognition that AI can be designed to have (or can later develop) racist or sexist attitudes and behaviours, students will need to be taught to be ever-vigilant for this type of prejudice.

Various suggestions have been made to reduce bias, including the development of tools and models such as PROBAST ( Wolff *et al.*, 2019), and medical practitioners will also need to be cognisant of data usage and security laws, such as GDPR, the 21st Century Cures Act ( USA Hse of Rep, 2016) and Chapter V of the Federal Food, Drug and Cosmetic Act ( FDA, 2018). Further discussion of the applicable regulations can be found in ( Pesapane *et al.*, 2018).

As the arguments about ethics become arguments about laws, the issues become more complex. When AI disagrees with humans, who takes precedence, and on what grounds? If a doctor defers to a recommendation by an AI system, does that mean that a portion of the medical responsibility has been ceded? Does this also mean that a portion of the doctors’ rights have been ceded, and, if not, why not? Related to the responsibility, when the AI makes mistakes, who is legally responsible? Apart from the fact that legalities will differ across the world, this issue is still unresolved, and, currently, the answer remains “It Depends” ( Jha, 2020). If an AI system can be found “guilty”, then does that pre-suppose legal rights? The notion of legal rights for AI and AI robots is not as ludicrous as one might first believe, and are currently being discussed ( Easen, 2019).

It is crucial, therefore, that medical students are taught to consider these questions.

This paper has considered much that will need to be put into the curriculum. It is a truism, however, that medical curricula are already overloaded; adding more content would appear to over-burden the staff and students. The difficult task will be removing items from the curriculum as these new items are inserted. For this, human and AI curriculum designers will have to work carefully, and the removal will depend on the circumstances of training and practice.

One should remember, though, that there is precedent. Over the years, less-prevalent diseases, and less-likely symptoms have been removed from curricula to be replaced by more-prevalent diseases and more-likely symptoms; old medical procedures have been replaced by new. So, too, old methods of diagnosis and treatment will need to be replaced by new AI-supported methods. One may ask with some fear, “What, then, if there is no AI system available?” I would estimate that that is the same question that might have been asked when the stethoscope was first introduced: “What if we train students to use a stethoscope instead of the old method, and then they lose their stethoscope?” We understand that there is this risk, and we do what we can to mitigate it.

## Conclusion

This paper has presented a conceptual and practical framework of AI in medical education, examining both administrative concerns and AI-related content to be included in the medical curriculum. In doing so, it has given details of many AI projects so that all medical education stakeholders (students, teachers and administrators) may be aware of current developments. Simultaneously, however, the framework and the related issues form a context and a guide for future work, not only for those who are developing AI systems, but for those who need to ensure that future medical practitioners are well-equipped to deal with the complexities and possibilities presented to them in the AI medical world.

## Take Home Messages

• Artificial Intelligence (AI) will have an impact on medical education.
• There are many current AI projects in education, and many more to come.
• It is necessary to have some conceptual and practical framework for these projects so that we can understand the overall context, future needs, and be able to respond appropriately to those needs.
• This paper provides such a framework, and gives details of the needs and possibilities of the framework’s components.

## Notes On Contributors

Ken Masters (PhD, FDE) is Associate Professor of Medical Informatics in the Department of Medical Education and Informatics, Sultan Qaboos University, Oman. He teaches introductory concepts of AI to medical students as part of a larger Medical Informatics course. He is the author and co-author of several papers dealing with informatics in medical education, including the *AMEE Guide to AI in Medical Education.* ORCID: https://orcid.org/0000-0003-3425-5020.

## Declarations

The author is an Associate Editor of MedEdPublish. This paper has been submitted through the standard MedEdPublish process.

## References

[ref1] Acikkar M. and Akay M. F. (2009) Support vector machines for predicting the admission decision of a candidate to the School of Physical Education and Sports at Cukurova University. Expert Systems with Applications. 36, pp.7228–7233. 10.1016/j.eswa.2008.09.007

[ref2] Afzal S. Dhamecha T. Gagnon P. Nayak A. (2020) AI Medical School Tutor: Modelling and Implementation.in Artificial Intelligence in Medicine. 18th International Conference on Artificial Intelligence in Medicine, AIME 2020, August 25-28, 2020. Minneapolis: Springer, pp.133–159. 10.1007/978-3-030-59137-3_13

[ref3] Agaoglu M. (2016) Predicting Instructor Performance Using Data Mining Techniques in Higher Education. IEEE Access. 4, pp.2379–2387. 10.1109/ACCESS.2016.2568756

[ref4] Alban M. and Mauricio D. (2019) Neural Networks to Predict Dropout at the Universities. International Journal of Machine Learning and Computing. 9(2), pp.149–153. 10.18178/ijmlc.2019.9.2.779

[ref5] Alemdag E. and Cagiltay K. (2018) A systematic review of eye tracking research on multimedia learning. Computers and Education. 125(June), pp.413–428. 10.1016/j.compedu.2018.06.023

[ref6] Alonso-Silverio G. A. Pérez-Escamirosa F. Bruno-Sanchez R. Ortiz-Simon J. L. (2018) Development of a laparoscopic box trainer based on Open Source hardware and Artificial Intelligence for objective assessment of surgical psychomotor skills. Surgical Innovation. 25(4), pp.380–388. 10.1177/1553350618777045 29809097

[ref7] Andris C. Cowen D. and Wittenbach J. (2013) Support Vector Machine for Spatial Variation. Transactions in GIS. 17(1), pp.41–61. 10.1111/j.1467-9671.2012.01354.x

[ref8] Anh N. X. Nataraja R. M. and Chauhan S. (2020) Towards near real-time assessment of surgical skills: A comparison of feature extraction techniques. Computer Methods and Programs in Biomedicine. 187, pp.105234–105234. 10.1016/j.cmpb.2019.105234 31794913

[ref9] Ashraf H. Sodergren M. H. Merali N. Mylonas G. (2018) Eye-tracking technology in medical education: A systematic review. Medical Teacher. Informa UK Ltd. 40(1), pp.62–69. 10.1080/0142159X.2017.1391373 29172823

[ref10] Asogwa O. C. and Oladugba A. V. (2015) Of students academic performance rates using Artificial Neural Networks (ANNs). American Journal of Applied Mathematics and Statistics. 3(4), pp.151–155. 10.12691/ajams-3-4-3

[ref11] Babić I. Đ. (2017) Machine learning methods in predicting the student academic motivation. Croatian Operational Research Review. 8, pp.443–461.

[ref12] Baker M. Harrison I. and Muir Gray . (2006) Safer IT in a safer NHS: account of a partnership. The British Journal of Healthcare Computing & Information Management. 23(7), pp.11–14.

[ref13] Bakshi S. K. Lin S. R. Shu D. Ting W. (2020) The era of artificial intelligence and virtual reality: transforming surgical education in ophthalmology. Br J Ophthalmol. [In press]. 10.1136/bjophthalmol-2020-316845 32816750

[ref14] Becker H. S. and Geer B. (1958) The Fate of Idealism in Medical School. American Sociological Review. 23(1), p.50. 10.2307/2088623

[ref15] Bernacki M. L. Chaves M. M. and Uesbeck P. M. (2020) Predicting Achievement and Providing Support before STEM Majors Begin to Fai. Computers & Education. p. Pre-Proof. 10.1016/j.compedu.2020.103999

[ref16] Billinghurst M. and Savage J. (1996) Adding intelligence to the interface.in Virtual Reality Annual International Symposium. Santa Clara, CA: IEEE, pp.168–175.

[ref17] Bourlas P. Giakoumakis E. Koutsouris D. Papakonstantinou G. (1996) The CARDIO-LOGOS system for ECG training and diagnosis. Technology and Health Care. 3(4), pp.279–285. 8705403

[ref18] Caudell T. P. Summers K. L. Holten J. IV Hakamata T. (2003) Virtual patient simulator for distributed collaborative medical education. Anatomical Record - Part B New Anatomist. 270(1), pp.23–29. 10.1002/ar.b.10007 12526063

[ref19] Chary M. Parikh S. Manini A. F. Boyer E. W. (2019) A review of natural language processing in medical education. Western Journal of Emergency Medicine. 20(1), pp.78–86. 10.5811/westjem.2018.11.39725 30643605 PMC6324711

[ref20] Chaudhri V. K. Cheng B. Overtholtzer A. Roschelle J. (2013) Inquire Biology: A Textbook that Answers Questions. AI Magazine. 34(3), p.55. 10.1609/aimag.v34i3.2486

[ref21] Chen C.-K. (2010) Curriculum Assessment Using Artificial Neural Network and Support Vector Machine Modeling Approaches: A Case Study. IR Applications. 29, pp.1–23.

[ref22] Chen D. C. R. Kirshenbaum D. S. Yan J. Kirshenbaum E. (2012) Characterizing changes in student empathy throughout medical school. Medical Teacher. 34(4), pp.305–311. 10.3109/0142159X.2012.644600 22455699

[ref23] Chi M. VanLehn K. Litman D. and Jordan P. (2011) Empirically evaluating the application of reinforcement learning to the induction of effective and adaptive pedagogical strategies. User Modeling and User-Adapted Interaction. 21(1-2), pp.137–180. 10.1007/s11257-010-9093-1

[ref24] Clancey W. J. (1983) GUIDON. Technical Report #9. Stanford, CA. Available at: https://files.eric.ed.gov/fulltext/ED242311.pdf( Accessed: 29 September 2020).

[ref25] Clarke A. C. (1980) Electronic Tutors. OMNI. 2(9), pp.76–96.

[ref26] Conner N. (2018) Chinese school uses facial recognition to monitor student attention in class. The Telegraph. May. Available at: https://www.telegraph.co.uk/news/2018/05/17/chinese-school-uses-facial-recognition-monitor-student-attention/( Accessed: 29 September 2020).

[ref27] Easen N. (2019) Rights for robots: why we need better AI regulation. Raconteur. Available at: https://www.raconteur.net/risk-management/legal-innovation-2019/robot-rights-ethics( Accessed: 26 September 2020).

[ref28] Eliot C. and Woolf B. P. (1995) An adaptive student centered curriculum for an intelligent training system. User Modeling and User-Adapted Interaction. 5(1), pp.67–86. 10.1007/BF01101802

[ref29] Entezarjou A. Bonamy A.-K. E. Benjaminsson S. Herman P. (2020) Human- Versus Machine Learning-Based Triage Using Digitalized Patient Histories in Primary Care: Comparative Study. JMIR Medical Informatics. 8(9), p. e18930. 10.2196/18930 32880578 PMC7499160

[ref30] FDA (2018) FD&C Act Chapter V: Drugs and Devices. FD&C Act. Available at: https://www.fda.gov/regulatory-information/federal-food-drug-and-cosmetic-act-fdc-act/fdc-act-chapter-v-drugs-and-devices( Accessed: 29 September 2020).

[ref31] Frize M. and Frasson C. (2000) Decision-support and intelligent tutoring systems in medical education. Clin Invest Med. 23(4), pp.266–269. 10981539

[ref32] García-Gorrostieta J. M. López-López A. and González-López S. (2018) Automatic argument assessment of final project reports of computer engineering students. Computer Applications in Engineering Education. 26(5), pp.1217–1226. 10.1002/cae.21996

[ref33] Gierl M. J. Latifi S. Lai H. Boulais A.-P. (2014) Automated essay scoring and the future of educational assessment in medical education. Medical Education. 48(10), pp.950–962. 10.1111/medu.12517 25200016

[ref34] Gijsberts C. M. Groenewegen K. A. Hoefer I. E. Eijkemans M. J. C. (2015) Race/Ethnic Differences in the Associations of the Framingham Risk Factors with Carotid IMT and Cardiovascular Events. PLoS One. 10(7), pp.e0132321–e0132321. 10.1371/journal.pone.0132321 26134404 PMC4489855

[ref35] Hamdy H. Al-Moslih A. Tavarnesi G. and Laus A. (2017) Virtual patients in problem-based learning. Medical Education. 51(5), pp.557–558. 10.1111/medu.13293 28295496

[ref36] Harden R. M. (2001) AMEE Guide No. 21: Curriculum mapping: A tool for transaprent and authentic teaching and learning. Medical Teacher. 23(2), pp.123–137. 10.1080/01421590120036547 11371288

[ref37] Hayasaka Y. Fujikura T. and Kashimura M. (2018) Expectations for the next generation of simulated patients born from thoughtful anticipation of artificial intelligence-equipped robot. Journal of Nippon Medical School. 85(6), pp.347–349. 10.1272/jnms.JNMS.2018_85-57 30568063

[ref38] Hershkovitz A. Baker R. Gowda S. and Corbett A. (2013) Predicting Future Learning Better Using Quantitative Analysis of Moment-by-Moment Learning.in EDM.

[ref39] Hoffait A.-S. and Schyns M. (2017) Early detection of university students with potential difficulties. Decision Support Systems. 101, pp.1–11. 10.1016/j.dss.2017.05.003

[ref40] Hojat M. Vergare M. J. Maxwell K. Brainard G. (2009) The Devil is in the third year: A longitudinal study of erosion of empathy in medical school. Acad Medicine. 84(9), pp.1182–1191. 10.1097/ACM.0b013e3181b17e55 19707055

[ref41] Hussain M. Zhu W. Zhang W. and Abidi S. M. R. (2018) Student Engagement Predictions in an e-Learning System and Their Impact on Student Course Assessment Scores. Computational Intelligence and Neuroscience. 2018, pp.1–21. 10.1155/2018/6347186 PMC6189675 30369946

[ref42] Jayaraman P. P. Forkan A. R. M. Morshed A. Haghighi P. D. (2019) Healthcare 4.0: A review of frontiers in digital health. WIREs Data Mining and Knowledge Discovery.pp.e1350–e1350. 10.1002/widm.1350

[ref43] Jha S. (2020) Can you sue an algorithm for malpractice? It depends, STAT. Available at: https://www.statnews.com/2020/03/09/can-you-sue-artificial-intelligence-algorithm-for-malpractice/( Accessed: 24 September 2020).

[ref44] Jindal A. and Bansal M. (2020) Knowledge and Education about Artificial Intelligence among Medical Students from Teaching Institutions of India: A Brief Survey. MedEdPublish. 9(1). 10.15694/mep.2020.000200.1

[ref45] Jović J. Milić M. Cvetanović S. and Chandra K. (2019) Implementation of machine learning based methods in elearning systems.in The 10th International Conference on eLearning (eLearning-2019), 26-27 September. Belgrade, Serbia, pp.39–44.

[ref46] Kardan A. A. and Sadeghi H. (2013) A Decision Support System for Course Offering in Online Higher Education Institutes. International Journal of Computational Intelligence Systems. 6(5), pp.928–942. 10.1080/18756891.2013.808428

[ref47] Kashou A. May A. DeSimone C. and Noseworthy P. (2020) The essential skill of ECG interpretation: How do we define and improve competency? Postgraduate Medical Journal. 96(1133), pp.125–127. 10.1136/postgradmedj-2019-137191 31874907

[ref48] Khin N. N. and Soe K. M. (2020) University Chatbot using Artificial Intelligence Markup Language.in 2020 IEEE Conference on Computer Applications(ICCA), Yangon, Myanmar, 2020. IEEE, pp.1–5. 10.1109/ICCA49400.2020.9022814

[ref49] Kintsch W. (2002) The potential of latent semantic analysis for machine grading of clinical case summaries. Journal of Biomedical Informatics. 35(1), pp.3–7. 10.1016/S1532-0464(02)00004-7 12415721

[ref50] Kolachalama V. B. and Garg P. S. (2018) Machine learning and medical education. npj Digital Medicine. Springer US,1(1), pp.2–4. 10.1038/s41746-018-0061-1 31304333 PMC6550167

[ref51] Laleye F. de Chalendar G. Blanié A. Brouquet A. (2020) A French Medical Conversations Corpus Annotated for a Virtual Patient Dialogue System.in Proceedings of the 12th Conference on Language Resources and Evaluation (LREC 2020), Marseille, 11-16 May 2020. European Language Resources Association, pp.574–580.

[ref52] Latifi S. Gierl M. J. Boulais A. P. and De Champlain A. F. (2016) Using automated scoring to evaluate written responses in English and French on a high-stakes clinical competency examination. Evaluation and the Health Professions. 39(1), pp.100–113. 10.1177/0163278715605358 26377072

[ref53] Liu Y. Chen P.-H. C. Krause J. and Peng L. (2019) How to Read Articles That Use Machine Learning: Users’ Guides to the Medical Literature. JAMA. 322(18), pp.1806–1816. 10.1001/jama.2019.16489 31714992

[ref54] Macrae H. Cohen M. Hashimoto D. and Miller B. (2020) Innovation in Surgical Education Technologies: COVID-19 and Beyond.in AMEE 2020: The Virtual Conference. AMEE 2020. Glasgow, Scotland: AMEE.

[ref55] Masters K. (2017) Preparing medical students for the e-patient. Medical Teacher. 39(7), pp.681–685. 10.1080/0142159X.2017.1324142 28532256

[ref56] Masters K. (2019) Artificial Intelligence in Medical Education. Medical Teacher. 41(9), pp.976–980. 10.1080/0142159X.2019.1595557 31007106

[ref57] McFadden P. and Crim A. (2016) Comparison of the effectiveness of interactive didactic lecture versus online simulation-based CME programs directed at improving the diagnostic capabilities of primary care practitioners. Journal of Continuing Education in the Health Professions. 36(1), pp.32–37. 10.1097/CEH.0000000000000061 26954243

[ref58] Michael J. Rovick A. Glass M. Zhou Y. (2003) Learning from a computer tutor with Natural Language capabilities. Interactive Learning Environments. 11(3), pp.233–262. 10.1076/ilee.11.3.233.16543

[ref59] Mirchi N. Bissonnette V. Yilmaz R. Ledwos N. (2020) The virtual operative assistant: An explainable artificial intelligence tool for simulation-based training in surgery and medicine. PLoS One. 15(2), pp.1–15. 10.1371/journal.pone.0229596 PMC7046231 32106247

[ref60] Mosier K. L. and Skitka L. J. (1996) Human Decision Makers and Automated Decision Aids: Made for Each Other?in Parasuraman R. and Mouloua M. (eds) Automation and Human Performance: Theory and Applications. Mahwah, New Jersey: Lawrence Erlbaum, pp.201–220.

[ref61] Nagendran M. Chen Y. Lovejoy C. A. Gordon A. C. (2020) Artificial intelligence versus clinicians: Systematic review of design, reporting standards, and claims of deep learning studies in medical imaging. The BMJ. 368( March), pp.1–14. 10.1136/bmj.m689 PMC7190037 32213531

[ref62] Natarajan S. and Nasiripour S. (2019) Viral Tweet About Apple Card Leads to Goldman Sachs Probe. Bloomberg. Available at: https://www.bloomberg.com/news/articles/2019-11-09/viral-tweet-about-apple-card-leads-to-probe-into-goldman-sachs( Accessed: 26 September 2020).

[ref63] National Research Council (2015) Training Students to Extract Value from Big Data: Summary of a Workshop. Washington, DC: The National Academies Press. 10.17226/18981 26065052

[ref64] Neumann M. Edelhäuser F. Tauschel D. Fischer M. R. (2011) Empathy decline and its reasons: A systematic review of studies with medical students and residents. Academic Medicine. 86(8), pp.996–1009. 10.1097/ACM.0b013e318221e615 21670661

[ref65] Nirschl J. J. Janowczyk A. Peyster E. G. Frank R. (2018) A deep-learning classifier identifies patients with clinical heart failure using whole-slide images of H&E tissue. PLoS One. Edited by A. Marsden,13(4), p.e0192726. 10.1371/journal.pone.0192726 29614076 PMC5882098

[ref66] Oancea B. Dragoescu R. and Ciucu S. (2013) Predicting students’ results in higher education using neural networks.in International Conference on Applied Information and Communication Technology. Jelgava, Latvia, pp.190–193.

[ref67] O’Neill N. (2017) China destroys sassy bots after they bash communism. New York Post. August. Available at: https://nypost.com/2017/08/04/china-destroys-sassy-bots-after-they-bash-communism/amp/( Accessed: 29 September 2020).

[ref68] Oquendo Y. A. Riddle E. W. Hiller D. Blinman T. A. (2018) Automatically rating trainee skill at a pediatric laparoscopic suturing task. Surgical Endoscopy. Springer US,32(4), pp.1840–1857. 10.1007/s00464-017-5873-6 29071419 PMC5845064

[ref69] Papa F. and Shores J. (1992) Expert Systems Based Clinical Assessment and Tutorial Project. Fort Worth, TX.

[ref70] Parapadakis D. (2020) Can artificial intelligence help predict a learner’s needs? Lessons from predicting student satisfaction. London Review of Education. 18(2), pp.178–195. 10.14324/lre.18.2.03

[ref71] Parikh R. B. Teeple S. and Navathe A. S. (2019) Addressing Bias in Artificial Intelligence in Health Care. JAMA. 10.1001/jama.2019.18058 31755905

[ref72] Pesapane F. Volonté C. Codari M. and Sardanelli F. (2018) Artificial intelligence as a medical device in radiology: ethical and regulatory issues in Europe and the United States. Insights into imaging. 2018/08/15 edn,9(5), pp.745–753. 10.1007/s13244-018-0645-y 30112675 PMC6206380

[ref73] Randhawa G. K. and Jackson M. (2020) The role of artificial intelligence in learning and professional development for healthcare professionals. Healthcare Management Forum. 33(1), pp.19–24. 10.1177/0840470419869032 31802725

[ref74] Rani S. and Kumar P. (2017) A Sentiment Analysis System to Improve Teaching and Learning. Computer. 50(5), pp.36–43. 10.1109/MC.2017.133

[ref75] Salles A. Evers K. and Farisco M. (2020) Anthropomorphism in AI. AJOB Neuroscience. 11(2), pp.88–95. 10.1080/21507740.2020.1740350 32228388

[ref76] Schwab K. (2015) The Fourth Industrial Revolution: What it means and how to respond. Foreign Affairs. 151(December). Available at: https://www.foreignaffairs.com/articles/2015-12-12/fourth-industrial-revolution( Accessed: 23 September 2020).

[ref77] Sharples M. Jeffery N. Teather D. Teather B. (1997) A socio-cognitive engineering approach to the development of a knowledge-based training system for neuroradiology.in Du Boulay B. and Mizoguchi R. (eds) Artificial Intelligence in Education: Knowledge and Media in Learning Systems. Proceedings of AI-ED 97, 8th world conference, Kobe, Japan, August 18-22, 1997. Kobe, Japan: IOS Press, pp.402–409.

[ref78] Singh M. (2018) An Introduction to Artificial Intelligence. Delhi: Lakshay Books.

[ref79] Sit C. Srinivasan R. Amlani A. Muthuswamy K. (2020) Attitudes and perceptions of UK medical students towards artificial intelligence and radiology: a multicentre survey. Insights Into Imaging. 11(1), pp.14–14. 10.1186/s13244-019-0830-7 32025951 PMC7002761

[ref80] Skorikov M. and Momen S. (2020) Machine learning approach to predicting the acceptance of academic papers.in The 2020 IEEE International Conference on Industry 4.0, Artificial Intelligence, and Communications Technology (IAICT), Bali, July 7-9, 2020. Bali: IEEE, pp.113–117.

[ref81] Srinivas S. and Rajendran S. (2019) Topic-based knowledge mining of online student reviews for strategic planning in universities. Computers & Industrial Engineering. 128, pp.974–984. 10.1016/j.cie.2018.06.034

[ref82] Stasiu R. K. Britto J. de Dias J. da S. and Scalabrin E. (2001) Electrocardiogram Interpretation Guided by a Tutorial Expert.in 14th IEEE Symposium on Computer-Based Medical Systems. Bethesda, MD: IEEE, pp.487–492.

[ref83] Straw I. (2020) The automation of bias in medical Artificial Intelligence (AI): Decoding the past to create a better future. Artificial Intelligence in Medicine.p.101965. 10.1016/j.artmed.2020.101965 33250145

[ref84] Toivonen T. Jormanainen I. and Tukiainen M. (2019) Augmented intelligence in educational data mining. Smart Learning Environments. 6(1), p.10. 10.1186/s40561-019-0086-1

[ref85] Treceño-Fernández D. Calabia-del-Campo J. Bote-Lorenzo M. L. Gómez-Sánchez E. (2020) Integration of an intelligent tutoring system in a magnetic resonance simulator for education: Technical feasibility and user experience. Computer Methods and Programs in Biomedicine. [In press]. 10.1016/j.cmpb.2020.105634 32645627

[ref86] Ullah F. Sepasgozar S. Tahmasebinia F. Sepasgozar S. M. E. (2020) Examining the impact of students’ attendance, sketching, visualization, and tutors experience on students’ performance: A case of building structures course in construction management. Construction Economics and Building. 20(3), pp.78–102. 10.5130/AJCEB.v20i3.7056

[ref87] USA Hse of Rep (2016) 21st Century Cures Act (Public Law 114-225, 114th Congress). Available at: https://www.congress.gov/bill/114th-congress/house-bill/34( Accessed: 29 September 2020).

[ref88] Vincent J. (2016) Twitter taught Microsoft’s AI chatbot to be a racist asshole in less than a day. The Verge. Available at: https://www.theverge.com/2016/3/24/11297050/tay-microsoft-chatbot-racist( Accessed: 29 September 2020).

[ref89] Wattanapisit A. Teo C. H. Wattanapisit S. Teoh E. (2020) Can mobile health apps replace GPs? A scoping review of comparisons between mobile apps and GP tasks. BMC Medical Informatics and Decision Making. 20(1), pp.5–5. 10.1186/s12911-019-1016-4 31906985 PMC6945711

[ref90] Weidenbach M. Trochim S. Kreutter S. Richter C. (2004) Intelligent training system integrated in an echocardiography simulator. Computers in Biology and Medicine. 34(5), pp.407–425. 10.1016/S0010-4825(03)00084-2 15145712

[ref91] Wijayarathna G. K. and Zary N. (2019) Feasibility in using de-identified patient data to enrich artificial applications in medical education. EDULEARN19 Proceedings. 1(July), pp.7598–7604. 10.21125/edulearn.2019.1837

[ref92] Wilkinson M. D. Dumontier M. Aalbersberg I. J. J. Appleton G. (2016) The FAIR Guiding Principles for scientific data management and stewardship. Scientific Data. 3, pp.160018–160018. 10.1038/sdata.2016.18 26978244 PMC4792175

[ref93] Wolff R. F. Moons K. G. M. Riley R. D. Whiting P. F. (2019) PROBAST: A tool to assess the risk of bias and applicability of prediction model studies. Annals of Internal Medicine. 170(1), pp.51–58. 10.7326/M18-1376 30596875

[ref94] Wu D. Xiang Y. Wu X. Yu T. (2020) Artificial intelligence-tutoring problem-based learning in ophthalmology clerkship. Annals of Translational Medicine. 8(11), pp.700–706. 10.21037/atm.2019.12.15 32617320 PMC7327320

[ref95] Wu J. T. Wong K. C. L. Gur Y. Ansari N. (2020) Comparison of Chest Radiograph Interpretations by Artificial Intelligence Algorithm vs Radiology Residents. JAMA Network Open. 3(10), p. e2022779. 10.1001/jamanetworkopen.2020.22779 33034642 PMC7547369

[ref96] van der Niet A. G. and Bleakley A. (2020) Where medical education meets artificial intelligence: “Does technology care?”. Medical Education.( February), pp.1–7. 10.1111/medu.14131 32078175

[ref97] Yang Y. Y. and Shulruf B. (2019) An expert-led and artificial intelligence system-assisted tutoring course to improve the confidence of Chinese medical interns in suturing and ligature skills: A prospective pilot study. Journal of Educational Evaluation for Health Professions. 16, pp.1–8. 10.3352/jeehp.2019.16.7 30986892 PMC6517322

[ref98] Zhang J. Zhang P. and Xu B. (2019) Analysis of College Students’ Public Opinion Based on Machine Learning and Evolutionary Algorithm. Complexity. 2019, pp.1–10. 10.1155/2019/1712569

