# Supplementary material for: Artificial Intelligence developments in medical education: a conceptual and practical framework
Source: MedEdPublish (2016). 2020 Oct 26;9:239. [Version 1] doi: 10.15694/mep.2020.000239.1 (PMC10697470; doi:10.15694/mep.2020.000239.1)
Supplement: Peer review history

# Reviewer report: Reviewer response for version 1

**DOI:** 10.21956/mep.20200.r31263

**Published:** 2021-01-15

**Authors:** Barbara Jennings

**Affiliations:** 1 Norwich Medical School

This review has been migrated. The reviewer awarded 5 stars out of 5 My advice to anyone who thinks about medical education is that you must read this article. The writing is crystal-clear and it is an enjoyable read. I bet it will provoke you to rethink whatever aspect of your curriculum you work on and to read a lot more about Artificial Intelligence/AI (or at least to plan to). Completing said reading will be easy because of the extensive and interesting bibliography included.The introduction calls for a needs-assessment to decide how AI is best applied to your context – and the author’s proposed logical framework for medical education subdivides issues into two broad categories: curriculum content and administration & methodology. The importance of AI literacy to a modern medical curriculum and our responsibility to train AI-enabled doctors is well-articulated throughout this paper.The individual issues described in the framework-map were explored by the author - and along the way AI principles, opportunities, and challenges were described. Some of the topics elaborated on, were personalised curriculum maps; non-invasive ways to track student participation and feedback; student-prompted systems for accessing advisory and counselling services without bureaucracy; adaptive instruction and the notion of the electronic teacher (it strikes me that we are all e-teachers, since covid-19 thundered into our lives!) But the topic that turned my head in particular, was the potential for case based learning (see the Teaching section of the article). The application of sound educational principles to an exploration of AI and the useful framework presented for medical education - makes this an article that I will be recommending highly to curriculum leads, instructional designers, teachers, and students.

Reviewer Expertise:

NA

No decision status is available

# Reviewer report: Reviewer response for version 1

**DOI:** 10.21956/mep.20200.r31261

**Published:** 2020-12-10

**Authors:** Hossam Hamdy

**Affiliations:** 1 Gulf Medical University

This review has been migrated. The reviewer awarded 5 stars out of 5 Artificial Intelligence (AI) in medical education is used without a clear or unified definition of its meaning. This article and the BEME systematic review written by Ken Masters present a conceptual and practical framework for understanding, developing, and applying AI in educational management, teaching, learning and medical practice. It provides practical examples on how AI can be used in curriculum design and management, curriculum mapping and in quality assurance. It can play a significant role in preparing program self-studies and used for accreditation. Introducing AI as a course or integrated in all aspects of the curriculum is taking place in different Health Professional Programs and will be a key feature of medical education. How to do it is related to the context of the culture and health system. What to teach and how are well described in the article. Of course, “One size does not fit all”.

Reviewer Expertise:

NA

No decision status is available

# Reviewer report: Reviewer response for version 1

**DOI:** 10.21956/mep.20200.r31264

**Published:** 2020-12-08

**Authors:** Martin Pusic

**Affiliations:** 1 Harvard Medical School

This review has been migrated. The reviewer awarded 5 stars out of 5 Dr. Masters presents an overview for undergraduate medical educators who wish to make their curricula AI aware and AI updated. He separates the considerations nicely, in Figure 1, by whether the consideration is about the Curriculum or the Structure of the Medical School. This can serve as an organizer for the leadership of a professional school.I would highlight the idea that the clinician of the future will be "engaged in a cooperative exercise with the (AI) tool" -- this touches on socio-material learning, a pedagogic theory that has been perhaps underplayed to date, but will be brought to the foreground with the coming adjustment to the full range of AI. Educators will do well to think about AI both in the way we educate and the way we take care of patients. This paper is a good roadmap for the coming shift.

Reviewer Expertise:

NA

No decision status is available

# Reviewer report: Reviewer response for version 1

**DOI:** 10.21956/mep.20200.r31265

**Published:** 2020-12-08

**Authors:** John Sandars

**Affiliations:** 1 Edge Hill University

This review has been migrated. The reviewer awarded 5 stars out of 5 AI is ubiquitous to our lives - even though we may not realise it! This paper provides an excellent overview of AI and its implications for medical education, with a focus on the key competencies required for living and working in the new age of AI. There are important lessons for all involved on designing. delivering and implementing medical education.

Reviewer Expertise:

NA

No decision status is available

# Reviewer report: Reviewer response for version 1

**DOI:** 10.21956/mep.20200.r31262

**Published:** 2020-11-04

**Authors:** P Ravi Shankar

**Affiliations:** 1 American International Medical University

This review has been migrated. The reviewer awarded 5 stars out of 5 I enjoyed reading this article about the artificial intelligence (AI) developments in medical education. AI is going to play an increasingly important role in education and health care practice like in other fields of human endeavour. One of the challenges is that many medical doctors and educators at present do not know much about AI as mentioned by the author. The situation may be analogous to that of computers in the 1990s when most of us feared the rapid inroads made by computers while today nearly all of us use computers in one form or the other. AI will become more user-friendly and nearly of us will be using AI in medicine. In fact, we already do in many applications. Current student and curriculum management system can offer some of the features mentioned by the author and the others may follow. One of the big concerns is privacy where organizations can intensively monitor students and employees and strong data protection frameworks are required. Providing a personal study plan for students is an important advantage of AI and some institutions have begun offering this. Learning can take place in three dimensional virtual spaces and AI-based systems can support the human teacher. The author also raises some important questions on the social and intellectual status of doctors when AI is already able to outperform doctors in some areas. The number of these areas will increase as AI continues to rapidly develop. The author has recommended some further resources for teachers interested in AI. What will be the best method for medical school faculty to develop a working knowledge of AI and be ready to use it in education? What resources and courses are currently available?

Reviewer Expertise:

NA

No decision status is available
